# 3D kidney segmentation from abdominal diffusion MRI using an appearance-guided deformable boundary

**DOI:** 10.1371/journal.pone.0200082

**Published:** 2018-07-13

**Authors:** Mohamed Shehata, Ali Mahmoud, Ahmed Soliman, Fahmi Khalifa, Mohammed Ghazal, Mohamed Abou El-Ghar, Moumen El-Melegy, Ayman El-Baz

**Affiliations:** 1 Bioengineering Department, University of Louisville, Louisville, KY, United States of America; 2 Department of Electronics and Communications Engineering, Mansoura University, Mansoura, Egypt; 3 Electrical and Computer Engineering Department, Abu Dhabi University, Abu Dhabi, UAE; 4 Radiology Department, Urology and Nephrology Center, University of Mansoura, Mansoura, Egypt; 5 Department of Electrical Engineering, Assiut University, Assiut, Egypt; Sichuan University West China Hospital, CHINA

## Abstract

A new technique for more accurate automatic segmentation of the kidney from its surrounding abdominal structures in diffusion-weighted magnetic resonance imaging (DW-MRI) is presented. This approach combines a new 3D probabilistic shape model of the kidney with a first-order appearance model and fourth-order spatial model of the diffusion-weighted signal intensity to guide the evolution of a 3D geometric deformable model. The probabilistic shape model was built from labeled training datasets to produce a spatially variant, independent random field of region labels. A Markov-Gibbs random field spatial model with up to fourth-order interactions was adequate to capture the inhomogeneity of renal tissues in the DW-MRI signal. A new analytical approach estimated the Gibbs potentials directly from the DW-MRI data to be segmented, in order that the segmentation procedure would be fully automatic. Finally, to better distinguish the kidney object from the surrounding tissues, marginal gray level distributions inside and outside of the deformable boundary were modeled with adaptive linear combinations of discrete Gaussians (first-order appearance model). The approach was tested on a cohort of 64 DW-MRI datasets with b-values ranging from 50 to 1000 s/mm^2^. The performance of the presented approach was evaluated using leave-one-subject-out cross validation and compared against three other well-known segmentation methods applied to the same DW-MRI data using the following evaluation metrics: 1) the Dice similarity coefficient (DSC); 2) the 95-percentile modified Hausdorff distance (MHD); and 3) the percentage kidney volume difference (PKVD). High performance of the new approach was confirmed by the high DSC (0.95±0.01), low MHD (3.9±0.76) mm, and low PKVD (9.5±2.2)% relative to manual segmentation by an MR expert (a board certified radiologist).

## Introduction

The prevalence of chronic kidney disease (CKD) in the U.S. (2017) was about 15% of the adult population and 662,000 were of end-stage kidney disease (ESKD) [1]. Although transplantation is the definitive therapy for ESKD, approximately 17,000 transplants were annually performed during this time due to limited availability of transplantable kidneys [2]. Thus, all efforts should be employed to prolong the survival rate of these kidneys. However, acute rejection (AR) is one of the most serious barriers to transplanted kidneys. Therefore, clinicians are in a bad need of a fast, accurate, and reliable diagnostic tool to early detect AR for a higher chance of rescuing the transplanted kidney [3], [4]. An imaging modality, such as diffusion-weighted magnetic resonance imaging (DW-MRI) with the ability to provide both anatomical and functional information, expose patients to no radiation or contrast agent like other imaging modalities (e.g., computed topography (CT) and dynamic MRI), is critically needed to develop a computer-aided diagnostic (CAD) system for the early detection of AR post-transplantation [5]. DW-MRI allows mapping of water molecules’ diffusion process in biological tissues, in-vivo and non-invasively. These water molecule diffusion patterns are quantified by apparent diffusion coefficients (ADCs) and reveal details about tissue (e.g., kidney) status, either normal or diseased. In particular, healthy renal allografts provide higher ADC values than those with AR ones and thus facilitate the transplant status classification process [3], [4], [6], [7]. Segmentation of transplanted kidney from the surrounding abdominal structures and tissues is the key step to developing such a CAD system [8]. Thus, in literature several studies have focused their research on kidney segmentation. However, kidney segmentation from DW-MRI is very rare and most of it is performed by clinical research either manually or by an ROI tool [9], [10], [3], [4]. To the best of our knowledge, our group is the only group who started to automatically segment kidneys from DW-MRIs. Therefore, we will overview the related work devoted to kidney segmentation on other image modalities. Namely, we will review some of these studies that used different segmentation methods (e.g., threshold, region growing and graph cuts, and evolving deformable boundary) and different imaging modalities (e.g., CT and dynamic MRI).

With threshold methods, the kidney is segmented through studying the pixel intensity distribution in a certain region of interest (ROI). The ROI can be placed in a manual, semi-automatic or automatic manner. Giele et al. [11] presented a method for segmenting and registering the kidney from dynamic contrast-enhanced MRI (DCE-MRI). Their method involves manual insertion of a contour surrounding the kidney in one image with high contrast, then the kidney motion in other images is handled using a phase difference movement detection technique. However, it had a low accuracy and it compensated for the translation resulting from the motion without referring to the rotation, which was revisited in [12]. Mavromatis and Sequeira [13] presented a segmentation algorithm for medical tissues based on calculating texture directional maximums to segment a cancerous kidney from CT images. Priester et al. [14] segmented the renal transplant in MR images using two groups of images; acquired before and after the injection of the gadolinium-diethylenetriamine pentaacetic acid (DTPA) contrast agent. The average images of each group are subtracted and the output is thresholded resulting in a binary mask. Post-morpholigical processing is conducted to obtain the renal cross section. Giele [15] used a similar subtraction technique followed by thresholding for renal segmentation. Post-processing using morphological operations was used to close kidney contours. Separating the cortex from the medulla involves generating two ROIs (outer and inner) on the shape of onion rings using erosion. The outer ROI contains the cortex tissues and the inner ROI also contains the medulla tissues. They separated the calix manually. Koh et al. [16] used 3D H-maxima morphological transform for renal segmentation, where training data and prior information are being excluded by using edge information and rectangular masks.

Pohle and Toennies [17] segmented the kidney cortex using a region growing method. Their method is capable of self-learning of the homogeneity criterion based on the properties of the region under consideration. Boykov et al. [18] presented a globally optimal segmentation method for registered renal dynamic multidimensional data using temporal Markov based graph cuts. A vector having intensity values over time is used to characterize every voxel. Some seed points are initially inserted over the objects and the background, which are used to estimate a 2D histogram to be used with energy minimization. The properties of regions and boundaries are compromised to meet the imposed constraints. In spite of the good results of this technique, it requires a manual interaction from user. Rusinek et al. [19] used rigid registration with graph cut for renal segmentation in order to handle displacements. Farag et al. [20] presented a segmentation method for kidney that combined shape constraints with boundary properties and regions using graph cuts. Chevaillier et al. [21] segmented the internal kidney components in DCE-MRI images. They categorized the pixels based on the contrast evolution with time, making use of vector quantization algorithm. As in [18], their method requires interaction from the user. Freiman et al. [22] proposed an automatic, non-parametric graph min-cut model based technique for segmentation of kidney from CT scans. In their technique both shape and intensity information are integrated in the model. The latter is iteratively estimated using expectation maximization, which meanwhile performs segmentation through estimating the maximum a posteriori Markov random field using the graph min-cut. Li et al. [23] automatically segmented the kidneys using wavelet based clustering. Yang et al. [24] classified kidney tissues using fuzzy c-mean clustering.

Furthermore, kidney segmentation was explored using evolving deformable boundary techniques. Leventon et al. [25] combined the shape and deformable model by attracting the level-set function to the likely shapes from a training set specified by principal component analysis (PCA). Wang et al. [26] discussed using constrained optimization with deformable contours and applied it for kidney segmentation. Their system handled noise by using region information as constraints in addition to boundary information. Tsagaan et al. [27] presented a technique for 3D kidney segmentations from CT scan using a deformable model, which is described by a non-uniform rational B-spline (NURBS) surface and a priori shape. They used the principal curvature as a shape feature. Several research has been performed to segment kidneys using DCE-MRIs in humans and rats [28], [29]. For human studies, large scale movement for kidney registration and segmentation was roughly handled by Sun et al. in [30] using translational gradient based similarity registration. This was followed by the subtraction of an image with high contrast from a pre-contrast one, where the resulting difference was used to find the kidney contour by applying the level-set method. This contour was transferred across other frames to estimate the parameters of the registration. Concerning rats, kidney contours were found by Sun et al. [28] using a level-set variational method, integrating a model for subpixel movement and smoothness temporal constraints. The medulla and the cortex were segmented using the level-set method [31]. Using hybrid region- and edge-based models added an improvement to the segmentation techniques utilizing deformable models [32]. Moreover, multiple object segmentation was performed using multiphase level-sets [33], [34]. Based on both intensity and shape prior information, Abdelmunim et al. [35], [36] implemented a variational level-set segmentation framework to segment medical shapes (e.g., kidney). Spiegel et al. [37] presented a 3D kidney segmentation approach from CT scans using an active shape model in which non-rigid registration is used to find the correspondence between input training data points. A deformable model based parametric framework for segmenting kidneys was presented by Yuskel et al. [38]. The evolution of the contour had a shape prior constraint using signed distance maps, in addition to an intensity based distribution constraint calculated using linear combination of discrete Gaussians (LCDG) [39]. Campadelli et al. [40] proposed a framework for automatic segmentation of kidneys in CT images. Their framework is intensity based and depends on a multiplanar fast marching technique. The framework is generic and can be used for segmentation of various organs due to its robustness to intensity and shape variations. Gloger et al. [41] used Bayesian statistics in addition to shape prior to build a level-set kidney segmentation framework. Cuingnet et al. [42] presented an automatic method for detecting and segmenting kidneys in CT images. After kidneys localization, probabilistic kidney maps were calculated using random forests, utilizing both intensity and first/second order derivatives of each voxel and its neighbors. Finally, template deformation algorithm was performed on these maps to extract kidney surface.

Although the aforementioned segmentation techniques have relatively succeeded in segmenting kidneys with high contrast from CT and dynamic MRI, these techniques were not designed to handle challenges that exist in DW-MRIs such as low signal-to-noise ratio (SNR), low contrast, and diffused boundaries due to the high intensity similarities between the kidney and its background, especially at high b-values, which hinder the aforementioned methods from accurate segmentation of kidneys from diffusion MRIs.

**Fig 1:**
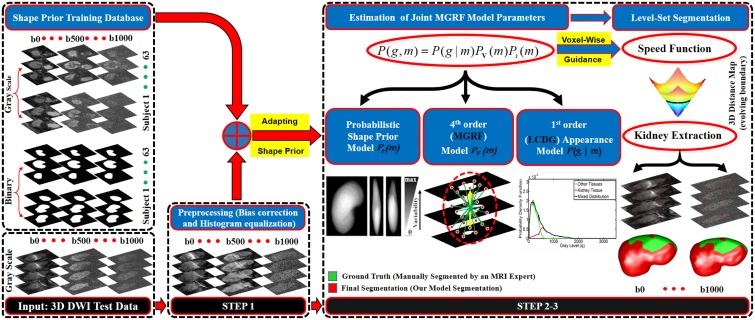
The proposed framework for kidney segmentation.

To overcome these limitations, this paper presents a DW-MRI geometric deformable kidney segmentation framework, shown in Fig 1, that is robust to noise and low contrast. This is achieved by combining higher-order Markov-Gibbs random field (MGRF) model parameters and adaptive shape model, in addition to the first-order visual appearance model, into a joint MGRF model. Our framework includes preprocessing using bias correction [43] and histogram equalization, estimation of the joint MGRF model parameters, and extraction of the kidney volume from the surrounding tissues using the level-sets guided by the estimated joint MGRF model. This paper presents the following specific contributions. (*i*) Higher order MGRF appearance model (up to the 4^*th*^-order), which takes into account the spatial dependencies between each voxel and its nearest neighbors in the DW-MR images. This better accounts for low SNR and low contrast, in addition to intra-kidney variabilities. (*ii*) An adaptive shape prior model of the expected kidney shape, which has the advantage over the fixed shape prior model not only to increase the robustness against noise and low contrast, but also to handle kidney motions caused by breathing and heart beating and to account for kidney variability due to inter-patient anatomical differences. This is achieved by adapting the shape probabilities of the kidney volume to be segmented based on its visual appearance. It is worth mentioning that the kidney-background visual appearance, shape prior, and statistical spatial dependencies are adaptable to kidney labels, which is an advantage of the presented framework.

## Materials and methods

### Participants

In total, 72 patients who underwent kidney transplantation provided verbal consents to participate in this study. All scans and biopsies were performed from July 2014 to February 2017, and all kidney transplants were done at Mansoura University (Egypt), with the donated kidneys having been obtained from live donors. However, eight patients were excluded due to a later on refusal of the study and/or contraindications for the MRI such as metallic prostheses, technical problems, artificial valves, or claustrophobia. The remaining 64 patients, (52 males and 12 females) and range in age from 12 to 54 years (the mean age of 27.2 ± 10.2 years), were included in the study. Patients were divided into two groups: non-rejection (NR) and acute rejection (AR). As a part of routine medical care after transplantation, all patients of both groups were assessed with serum creatinine laboratory values with a normal (basal) level of ≤ 1.3 *mg* ⋅ *dl*^−1^. The NR group (17 patients) included patients with healthy graft function. Most of the NR group patients only underwent the DW-MRI scans two weeks after transplantation. The AR group (47 patients) included patients with acute renal rejection, based on renal biopsy histology. All patients of the AR group underwent the DW-MRI scans two weeks after transplantation and just before the renal biopsy. All patients were asked to hold respiration (breath) during the study to reduce respiratory effect. Both the DW-MRI scans and biopsy were examined by a nephrologist and a board certified radiologist.

### Imaging protocol

The DW-MR images were acquired before any biopsy procedure by using a 1.5T SIGNA Horizon scanner (General Electric Medical Systems, Milwaukee, WI, USA). Coronal DW-MR images have been obtained by using a body coil and a multi-shot spin-echo echo-planar sequence (TR/TE, 8000/61.2 *ms*; bandwidth, 142 *kHz*; 1.28 × 1.28 *mm*^2^ matrix; section thickness of 4 *mm*; intersection gap of 0 *mm*; FOV of 36 *cm*; 7 acquired signals; water signals acquired at different *b*-values of (*b*_0_, *b*_50_, *b*_100_, *b*_200_, *b*_300_, *b*_400_, *b*_500_, *b*_600_, *b*_700_, *b*_800_, *b*_900_, and *b*_1000_) *s*/*mm*^2^) using a single-direction from right to left. Approximately 50 sections have been obtained in 60–120 seconds to cover the whole kidney.

### Detailed methods

Given an input (3D + *b*-value) DW-MRI, the presented segmentation technique in Fig 1 performs the following steps: (*i*) preprocessing of the DW-MRI kidney volume to be segmented; (*ii*) estimating of the joint Markov-Gibbs random field (MGRF) model parameters, namely, the adaptive shape model and the DW-MRI visual appearance features; and (*iii*) extracting the kidney volume from the surrounding tissues using the level-sets guided by the joint MGRF model estimated in the previous step.

**Fig 2:**
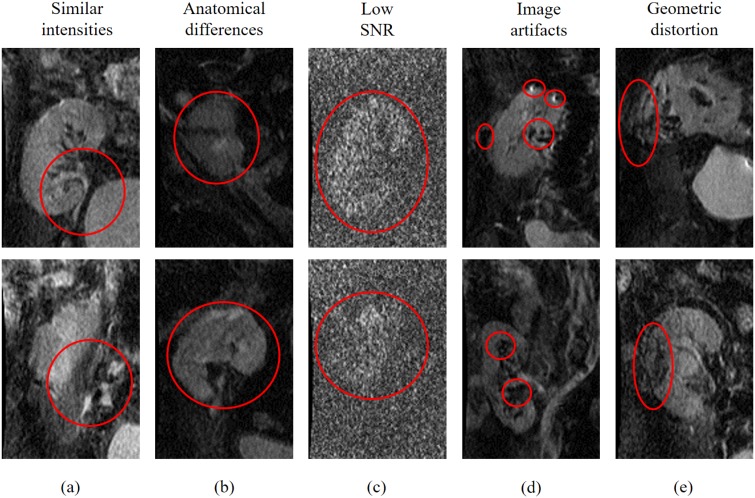
Typical coronal cross-section DW-MRI samples showing (a) low contrast between the kidney and surrounding abdominal tissues at *b*_0_; (b) inter-patient anatomical differences at *b*_0_, (c) low signal-to-noise ratio (SNR), especially, at higher *b*-values (e.g., *b*_1000_); (d) image artifacts; and (e) geometric distortion/diffused boundaries.

Achieving an accurate kidney segmentation is a challenging task [44], [38] because of kidney motions due to breathing and heart beating; kidney shape changes due to inter-patient anatomical differences; low contrast between the kidney and other abdominal structures, especially at higher gradient strengths and duration, or *b*-values (Fig 2); low SNR and artifacts that complicate image alignment [45]; and geometric distortions due to long acquisition time [46]. To overcome these challenges, our segmentation relies on multiple image features to accurately delineate the kidney and thus facilitates analysis of transplant status. Details of our segmentation pipeline and its basic notations are outlined below.

**Basic notations**: For describing processing steps, let **p** = (*x*, *y*, *z*) denote a voxel at 3D position with discrete Cartesian coordinates (*x*, *y*, *z*) and let **R** = {(*x*, *y*, *z*): 0 ≤ *x* ≤ *X* − 1; 0 ≤ *y* ≤ *Y*−1; 0 ≤ *z* ≤ *Z* − 1} be a finite 3D arithmetic lattice of unit voxels. The lattice has the size of *XYZ* and supports both grayscale images and their parametric or region (segmentation) maps. A grayscale image, **g** = {*g*_**p**_: **p** ∈ **R**; *g*_**p**_ ∈ **Q**}, takes voxel-wise values from a finite set, **Q** = {0, 1, …, *Q* − 1}, of *Q* integer gray levels, i.e. *g*: **R** → **Q**. A region map, **m** = {*m*_**p**_: **p** ∈ **R**; *m*_**p**_ ∈ **L**}, takes voxel-wise values from a binary set of region labels, **L** = {0, 1}, where 0 and 1 indicate the background and kidney, respectively, i.e. *m*: **R** → **L**.

**3D kidney segmentation**: The overall segmentation pipeline starts with a preprocessing step that combines histogram equalization with non-parametric bias correction [43] in which the noise and inconsistencies due to low-frequency non-uniformity, or inhomogeneity of intensities, are partially suppressed.

**Fig 3:**
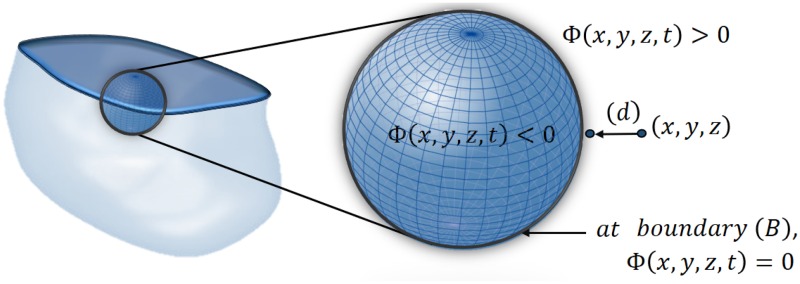
3D zero-level set of a function Φ(p = [*x*, *y*, *z*], *t*).

A 3D geometric (level-set-based) deformable boundary, is employed for the DW-MRI kidney segmentation due to successful results in a wide range of applications, including medical imaging, (e.g., for segmenting brain, prostate, liver, kidney etc.) [47], [48], [41], [5]. Due to simplicity, flexibility, and ability to handle complex shapes and topological changes independently of surface parameterizations, these deformable boundaries are more popular than the alternative parametric ones. Points of an object-background boundary at each time instant *t* are specified implicitly as a zero-level set, *B*_*t*_ = {**p**: **p** ∈ **R**; Φ(**p**, *t*) = 0}, of arguments of a specific higher-dimensional function, Φ(**p**, *t*), on the lattice **R**. The function is often a signed distance map:

$$\Phi \left(p,t\right)=\{\begin{array}{ccc} d(p,B_{t}) & \text{if} & p\;\;\text{is}\;\text{inside}\;\text{the}\;\text{boundary}\;B_{t};\\ 0 & \text{if} & p\;\;\text{is}\;\text{at}\;\text{the}\;\text{boundary}\;B_{t},\;\text{and}\\ -d(p,B_{t}) & \text{if} & p\;\;\text{is}\;\text{outside}\;\text{the}\;\text{boundary}\;B_{t} \end{array}$$ (1)

where $d\left(p,B_{t}\right)={\text{min}}_{b\in B_{t}}d\left(p,b\right)$ is the distance from the point **p** to the boundary *B*_*t*_, and *d*(**p**, **b**) is the Euclidean distance between two lattice points **p** and **b**, as shown in Fig 3. The function Φ(**p**, *t*) evolves in discrete time *t* = *nτ* with a fixed step, *τ* > 0, as [49]:

$$\Phi \left(p,\left(n+1\right)\tau \right)=\Phi \left(p,n\tau \right)-\tau F_{n}\left(p\right)\left|\nabla \Phi \left(p,n\tau \right)\right|$$ (2)

where *n* = 0, 1, 2, …, is the time index; ∇Φ(**p**, *nτ*) is the spatial gradient of Φ(**p**, *nτ*):

$$\nabla \Phi \left(p,n\tau \right)=[\frac{\partial \Phi (p,n\tau )}{\partial x},\frac{\partial \Phi (p,n\tau )}{\partial y},\frac{\partial \Phi (p,n\tau )}{\partial z}];$$ (3)

|**a**| denotes the magnitude of the vector **a**, and *F*_*n*_(**p**) is a speed function guiding the evolution of an initial boundary *B*_0_, defined at the starting instant *t* = 0, i.e., for *n* = 0.

Most of the conventional speed functions quantify visual appearance differences between the object and its background in terms of mean values and variances of image intensities, intensity edges, or gradient vector flow, and similar regional signal characteristics. Thus, their guidance may fail if images to be segmented are noisy and/or object-background contrast is low. To accurately segment the kidneys from noisy and low-contrast DW-MRI, our guiding function accounts for not only regional kidney-background appearance, but also for a kidney shape prior and high-order spatial relations in the goal region map. To provide voxel-wise guidance for the evolving boundary, all appearance and shape descriptors are combined into a joint Markov-Gibbs random field (MGRF) model of a DW-MR image, **g**, and its binary kidney-background region map, **m**. The model is specified by a joint probability distribution *P*(**g**, **m**) = *P*(**g**|**m**)*P*(**m**), where *P*(**g**|**m**) and *P*(**m**) denote a conditional probability distribution of images, given a map, and an unconditional distribution of region maps, respectively. The latter distribution is factored into two terms: *P*(**m**) = *P*_sp_(**m**)*P*_**V**_(**m**), where *P*_sp_(**m**) denotes an appearance-based adaptive shape prior, and *P*_**V**_(**m**) is a high-order Gibbs probability distribution with potentials **V**. The potentials evaluate strengths of not only the nearest-neighbor pairwise dependencies, but also of triple- and quadruple-dependencies, which specify a higher-order spatially homogeneous MGRF model of region maps. These components of the joint image-map model are outlined below.

#### First-order kidney/background appearance model

To accurately model DW-MRI appearance, we approximate the empirical marginal probability distribution of intensities with a linear combination of discrete Gaussians (LCDG) [39]. The LCDG with two positive dominant components (one each for the kidney and background) and multiple sign-alternate subordinate components allow for separating the mixed marginal of the DW-MRI voxel-wise intensities into the two distinct LCDGs, each associated with the kidney or background label. This LCDG model adapts the segmentation to changing appearance, such as non-linear intensity variations caused by patient weight and the data acquisition system, and it separates individual submodels of the kidney and background intensities more accurately than a conventional mixture of only positive Gaussians. This adaptation yields a better initial region map after the voxel-wise classification of only the image intensities with no account for the kidney shape.

#### Higher-order spatial interactions model

**Fig 4:**
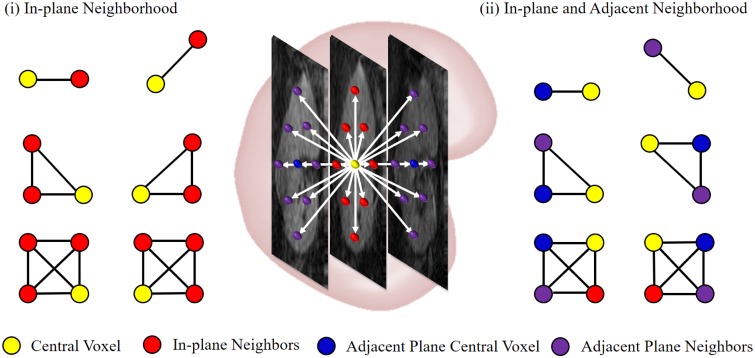
The nearest 26-neighborhood of a voxel for the 4^th^-order spatial model and examples of its 2^nd^-order cliques (upper raw), 3^rd^-order cliques (middle raw), and 4^th^-order cliques (lower raw). Note that the central voxel is shown in yellow, while its neighbors are shown (i) in red for the same plane and (ii) in blue and purple for the adjacent planes.

Compared to other imaging modalities, lower SNRs and frequent artifacts [45], together with geometric distortions due to long acquisition time [46] and larger inhomogeneities of internal structures, such as cortex and medulla, in the DW-MRI hinder the kidney segmentation. To better account for intra-kidney variabilities, spatial dependencies between each voxel and its nearest neighbors in the DW-MR images have been incorporated into our segmentation. Incorporated spatial relationships not only reduce noise impacts, but also reveal homogeneities and thus enhance the overall segmentation accuracy. Unlike the conventional pairwise-spatial homogeneity descriptors (e.g., in [50]), we use a 4^th^-order MGRF with analytically estimated potentials to describe those relationships. To find the potential estimates, an initial kidney map, **m**, is constructed by a simple Bayes classification using joint voxel-wise shape and intensity probabilities. Then, inter-label spatial dependencies in this map, **m**, are modelled by the 4^th^-order spatial MGRF with the nearest 26-neighborhood shown in Fig 4(a). This model adds triple and quadruple clique families to the more conventional 2^nd^-order Potts MGRF [50].

Let **C**_*a*_ be a family of *s*-order cliques of the interaction graph with nodes in the lattice sites **p** ∈ **R** and edges connecting interdependent pairs of the sites. Let *A* clique families describe spatial geometry of interdependencies of region labels in the kidney maps, **m**. Then the model is specified by the Gibbs probability distribution:

$$P_{V}\left(m\right)=\frac{1}{Z_{V}}\text{exp}(\sum_{a=1}^{A}\sum_{c\in C_{a}}V_{a}\left(m_{p}:p\in c\right))$$ (4)

where $V\;=\;[V_{a}{\left(\mu \right)\;:\;\mu ]\;in\;\left\{0,1\right\}}^{\nu_{a}}\;\to \;\left(-\infty ,\infty \right)\;:\;a\;=\;1,\ldots ,A]$ is a collection of potential functions for the families **C**_*a*_, *ν*_*a*_ is the clique size (*ν*_*a*_ ∈ {2, 3, 4}) for the family **C**_*a*_; ***μ*** is a label configuration on the clique, (i.e., a pair, triple, or quadruple of binary numbers 0 and 1), and *Z*_**V**_ is the normalizing factor, called the partition function, over the entire population $M={\left\{0,1\right\}}^{XYZ}$ of the maps:

$$Z_{V}=\sum_{m\in M}\text{exp}(\sum_{a=1}^{A}\sum_{c\in C_{a}}V_{a}\left(m_{p}:p\in c\right))$$ (5)

For equiprobable binary labels, *m*_**p**_ ∈ {0, 1}, the marginal co-occurrence probabilities over the 2^nd^-, 3^rd^-, and 4^th^-order cliques are $\frac{1}{4}$, $\frac{1}{8}$, and $\frac{1}{16}$, respectively. Provided the cardinalities of the clique families are close to the lattice cardinality for all the families *a* = 1, …, *A* and only the equality (“eq”) or inequality (“ne”) of all the clique-wise labels are taken into account, the corresponding estimates of the 2^nd^-, 3^rd^-, and 4^th^-order potentials are as follows:

$$V_{2:a:\text{eq}}=4(F_{a:\text{eq}}\left(m\right)-\frac{1}{2})=-V_{2:a:\text{ne}}$$ (6)

$$V_{3:a:{\text{eq}}_{3}}=\frac{16}{3}(F_{a:{\text{eq}}_{3}}\left(m\right)-\frac{1}{4})=-V_{3:a:{\text{eq}}_{2}}$$ (7)

$$V_{4:a:\text{eq}}=[\begin{array}{c} V_{4:a:{\text{eq}}_{4}}\\ V_{4:a:{\text{eq}}_{3}}\\ V_{4:a:{\text{eq}}_{2}} \end{array}]=\lambda^{*}[\begin{array}{c} f_{4:a}\\ f_{3:a}\\ f_{2:a} \end{array}]$$ (8)

where *F*_a:eq_(**m**) denote relative empirical frequencies of the equal binary labels in the cliques of each family **C**_*a*_ over a given training map **m**; “eq” and “ne” denote two equal or non-equal labels, respectively, for a 2^nd^-order clique; “eq_*i*_” denote *i* equal labels for a 3^rd^- and 4^th^-order clique; $f_{4:a}=F_{a:{\text{eq}}_{4}}\left(m\right)-\frac{1}{8}$; $f_{3:a}=F_{a:{\text{eq}}_{3}}\left(m\right)-\frac{1}{2}$; $f_{2:a}=F_{a:{\text{eq}}_{2}}\left(m\right)-\frac{3}{8}$; and

$$\lambda^{*}=\frac{\sum_{a=1}^{A}f_{4:a}^{2}+f_{3:a}^{2}+f_{2:a}^{2}}{\sum_{a=1}^{A}\frac{7}{64}f_{4:a}^{2}+\frac{1}{4}f_{3:a}^{2}+\frac{15}{64}f_{2:a}^{2}}$$ (9)

This approximation is used for computing the higher-order spatial probabilities Pr_**V**:**p**_(*l*) of each label; *l* ∈ **L**.

#### Adaptive shape prior model

In addition to the distinct visual appearances, the well-known geometric shapes of medical structures can enhance the segmentation accuracy. Relying on this fact, we use an adaptive model of the expected kidney shape to both handle kidney motions, (e.g., due to breathing and/or heart beating), and account for the kidney’s variability due to inter-patient anatomical differences. In addition, the kidney DW-MR images are very noisy, especially at high *b*-values.

**Fig 5:**
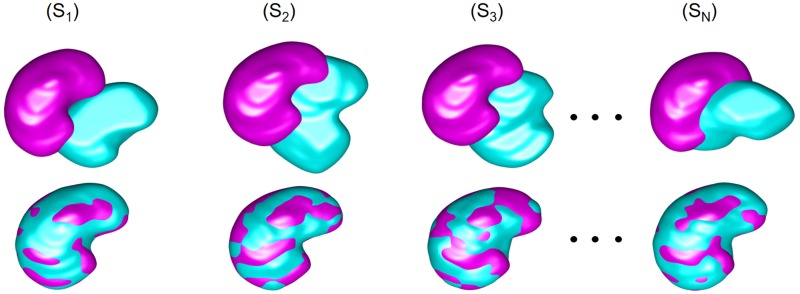
3D co-alignment of training DW-MRI datasets (*S*_1_:*S*_*N*_) to a single reference: The first and second rows present the overlapped 3D kidney volumes before and after the alignment, respectively. Note that the reference subject appears in magenta, while the targets are shown cyan.

To build the shape prior, all the *b*_0_-scans (after excluding the test subject) of kidneys formed a training database, and these images have been manually delineated by an MRI expert (a board certified radiologist) to get their binary kidney/background region maps. One of the images was chosen as a database reference. All other images were aligned to the reference by a non-rigid 3D registration [51] minimizing the sum of squared voxel-wise intensity differences between the two images. Then, the kidney/background labels of the co-aligned region maps were used to learn the shape prior. It is worth noting that to select the best reference subject, we performed a normalized cross-correlation between the test subject and every other subject in the shape prior training database. The subject with the maximum correlation was selected to be our reference. The choice of the reference has minimal effect on the shape prior as our presented shape prior depends on both the mapped spatial location in addition to signal appearance. In our presented shape prior, an adaptive search space around the mapped spatial location, which maps each voxel from the test subject to the database, is used in searching for voxels within a predefined tolerance range to the test voxel appearance. This means that any misalignment errors in the registration step will be overcome by the adaptive process. Moreover, the final segmentation takes into account the first-order appearance and the higher order spatial interaction that will overcome any errors from the shape prior segmentation. Fig 5 illustrates the co-alignment of the training DW-MRI. Adapting the shape prior to each input DW-MR image to be segmented is guided by the visual appearance of the latter image. The probabilistic shape prior is built as a spatially variant independent random field of region labels *P*_sp_(**m**) = ∏_**p**∈R_ Pr_**p**_(*m*_**p**_), where Pr_**p**_(*l*) is the marginal empirical probability of the label *l* ∈ **L** in the voxel **p**; ∑_*l*∈**L**_ Pr_**p**_(*l*) = 1.

As the DW-MR images are challenged by the motion, which might lead to different kidney masks at different *b*-values, each *b*-scan is segmented separately. The shape prior is built by segmenting manually and co-aligning the baseline scans (at *b*_0_ s/mm^2^) of all subjects. Then, the shape prior is applied to each *b*-scan in combination with the other estimated probabilistic models for that scan namely, the 1^*st*^-order LCDG model of current kidney appearance, in terms of voxel-wise intensities, and the 4^*th*^-order MGRF model of spatial interactions. This joint probabilistic model provides a stochastic force that guides the evolution of a deformable boundary to segment the kidneys at that *b*-scan. So, the segmentation of all *b*-value scans uses the same shape prior, but own intensity and spatial interactions models.

Algorithm 1 summarizes estimating and updating the appearance-guided shape prior for each test DW-MR image to be segmented (the test images are first removed from the training set).

**Algorithm 1** Creating/Updating the Shape Prior

1. Preprocess the training DW-MR images by bias correction and histogram equalization.

2. Construct the shape database by applying the co-alignment [51] to the preprocessed DW-MR images.

3. Preprocess the DW-MR image for a test subject and co-align with the shape database.

4. For each voxel, **p** ∈ **R**, in the test DW-MR image, **g**_test_, calculate its prior shape probabilities as follows:

 (a) Use the co-aligning deformation field to relate the voxel **p** of the test image to the shape database lattice.

 (b) Initialize a 3D window of size *N*_1_ × *N*_2_ × *N*_3_, centered at the related voxel in the shape database lattice.

 (c) Find within the window all the voxels with the corresponding intensity, *g*_test:**p**_, in all the training images.

 (d) If necessary, increase the window size and repeat Steps 4b to 4d until a non-empty set of such corresponding training intensities is found.

 (e) Estimate label probabilities based on relative occurrences of each label in all the training voxels found.

#### Appearance- and shape-guided deformable model

Adaptation to the kidney-background visual appearance, shape prior, and statistical spatial dependencies between kidney labels is one of the main advantages of our segmentation framework. Estimated directly from the input image and a given shape database, these properties guide the evolving deformable boundary by defining, for each voxel **p** with intensity *g*_**p**_ = *q*, the speed function [5] of Eq (2), *F*_*n*_(**p**) = *κϑ*_**p**_, where *κ* is the mean contour curvature and *ϑ*_**p**_ specifies the magnitude and direction of moving that voxel:

ϑp={-Prp(1)ifPrp(1)>Prp(0);i.e.,Prp(1)>0.5;Prp(0)otherwise (10)

Here, Pr_**p**_(0) and Pr_**p**_(1) are the voxel-wise background and kidney probabilities, respectively:

$$\underset{p}{\text{Pr}}\left(1\right)=\frac{\Omega_{\text{kd}:p}}{\Omega_{\text{kd}:p}+\Omega_{\text{bg}:p}};\;\;\underset{p}{\text{Pr}}\left(0\right)=\frac{\Omega_{\text{bg}:p}}{\Omega_{\text{kd}:p}+\Omega_{\text{bg}:p}}=1-\underset{p}{\text{Pr}}\left(1\right)$$ (11)

where the variables Ω_kd:**p**_ and Ω_bg:**p**_ for the kidney and background, respectively, depend on the voxel-wise probabilities Pr(*q*|*l*); *l* ∈ **L**, for the LCDG submodels of the kidney (*l* = 1) or background (*l* = 0) appearance and on the kidney label probability in the MGRF spatial region map model, Pr_**V**:**p**_(1), and in the adaptive shape prior, Pr_sp:**p**_(1), respectively:

$$\Omega_{\text{kd}:p}=\text{Pr}\left(q|1\right)\underset{V:p}{\text{Pr}}\left(1\right)\underset{\text{sp}:p}{\text{Pr}}\left(1\right);$$ (12)

$$\Omega_{\text{bg}:p}=\text{Pr}\left(q|0\right)(1-\underset{V:p}{\text{Pr}}\left(1\right)\left(1-\underset{\text{sp}:p}{\text{Pr}}\left(1\right)\right)$$ (13)

Algorithm 2 summarizes the basic steps of the 3D level-set-based kidney segmentation.

**Algorithm 2** DW-MRI Segmentation by Geometric Deformable Boundary

1. Approximate the marginal of DW-MRI intensities with the LCDG [39] with two dominant components.

2. Update the shape prior probability using Step 4 of Algorithm 1.

3. Form an initial region map, **m**_ini_, using the estimated shape prior and LCDG submodels of kidney and background appearances.

4. Estimate the Gibbs potentials for the 4^*th*^-order spatial MGRF map model from **m**_ini_.

5. Find the above speed function [5], *F*_*n*_(**p**), using results of Steps 1 to 4.

6. Segment the input image, **g**, by evolving the level-set function, Φ(**p**, *nτ*), of Eq (2) with the speed function found in Step 5.

## Results and discussion

**Fig 6:**
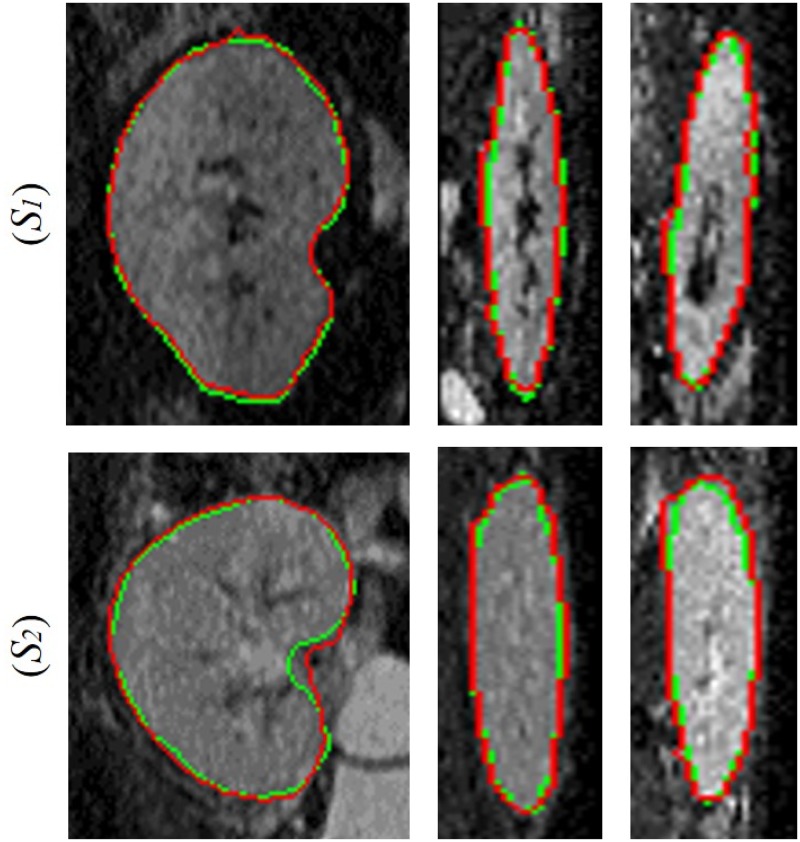
Our segmentation (red) with respect to the expert’s manual ground truth (green): The coronal (left), axial (middle), and sagittal (right) cross-sections for two different subjects in the first and second rows.

*Segmentation results*: The performance of the proposed segmentation approach was tested on the collected DW-MRI data (a total of 64 subjects). Fig 6 shows segmentation results for different kidney cross-sections (coronal, axial, and sagittal) for two subjects at *b*_0_. The segmentation accuracy was evaluated by two volumetric measures, namely, the Dice similarity coefficient (DSC) [52] and percentage kidney volume difference (PKVD), and one distance-based metric—the 95-percentile modified Hausdorff distance (MHD) [53], which characterize the spatial overlap and distribution of the surface to surface distances between the segmented and ground truth kidneys, respectively. The ground truth kidney maps were manually delineated by an MRI expert (a board certified radiologist).

**Fig 7:**
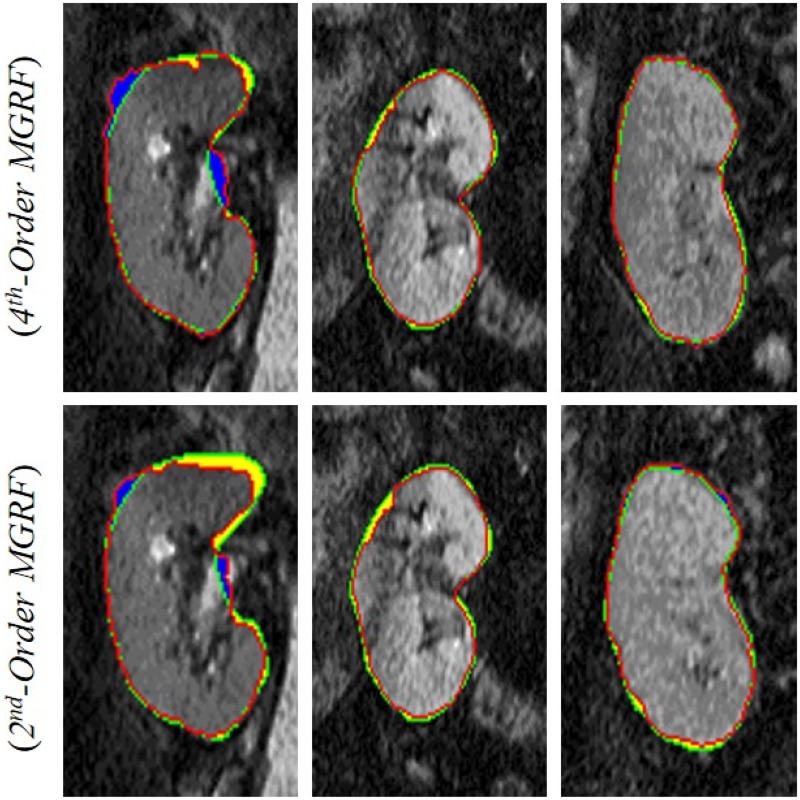
Our segmentation (red) with respect to the expert’s manual ground truth (green) using the 4^*th*^-order MGRF (first row) compared to our previous segmentation using the 2^*nd*^-order MGRF (second row) [54], [55] for three different subjects (columns), where the first, second, and third columns show large, moderate, and small differences in yellow regions (false positive (FP)) and blue regions (false negative (FN)), respectively.

To show the effect of adding the higher-order MGRF model to our segmentation, we compared the current results with our previous segmentation using the 2^*nd*^ order- MGRF [54], [55], as shown in Fig 7. Moreover, Table 1 compares both segmentation methods in terms of the DSC, MHD, and PKVD metrics. As shown in Fig 7 and documented in Table 1, our segmentation has been notably enhanced after adding the higher-order MGRF model. This can be explained in part by abilities of the latter model to capture more intricate inhomogeneities of grey levels between different structures, (e.g. cortex and medulla.)

**Table 1:** Our segmentation accuracy by the DSC, MHD (mm), and PKVD (%). All metrics are represented by the minimum (Min), maximum (Max), and mean_±_standard deviation (SD) values.

	Evaluation Metrics		
*Our method*	**DSC**	**MHD** (mm)	**PKVD** (%)
**Min**	0.92	2.5	5.7
**Max**	0.97	6.4	15.7
**Mean±SD**	0.95_±0.01_	3.9_±0.76_	9.5_±2.2_
*Previous* [54], [55]	**DSC**	**MHD** (mm)	**PKVD** (%)
**Min**	0.89	4.0	9.4
**Max**	0.96	8.0	21
**Mean±SD**	0.92_±0.02_	5.7_±2.0_	14_±3.1_
***P*-value**	<0.0001	<0.0001	<0.0001

**Fig 8:**
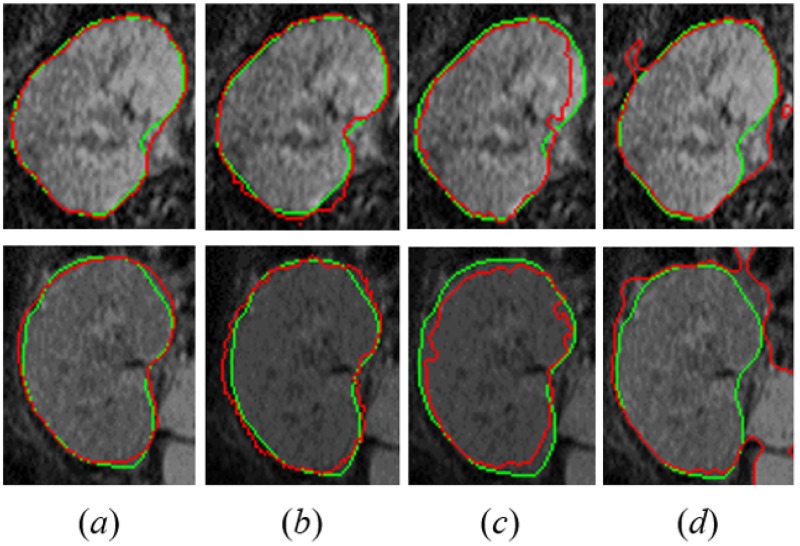
Comparative cross-sectional segmentation results for our approach (a), the vector level sets [35] (b), the segmentation using the Random Forest classifier [42] (c), and the traditional CV [31] level set (d) for two independent subjects (rows). The model segmentation is shown in red with respect to the manual ground truth (green) from an expert.

Moreover, the advantages of the presented segmentation approach are highlighted by comparing our results with those obtained from other three well-known segmentation approaches, namely, the vector level-sets [35] (VLS), the segmentation using the random forest classifier [42] (RFC), and the level-set by Chan and Vese [31] (CV). Fig 8 visually assesses the better segmentation accuracy of the proposed segmentation method compared to others [31], [35], [42] for two different subjects. Table 2 compares our segmentation method’s results with the results obtained from the previously mentioned methods in terms of the DSC, MHD, and PKVD metrics. As documented in Table 2, our segmentation performs much better than other segmentation methods by providing higher DSC, lower MHD, and lower PKVD values. Furthermore, our approach is confirmed, by the paired *t*-tests between the DSC, MHD, and PKVD values for our approach and the compared segmentation approaches, to have a statistically more significant performance than these approaches and evidenced by the *P*-values less than 0.05.

**Table 2:** Segmentation performance comparison between the presented approach against three other well-known segmentation methods (vector level-sets (VLS), random forest classifier (RFC), and Chan and Vese (CV)) using DSC, MHD (mm), and PKVD (%). All metrics are represented by the mean± standard deviation (SD).

	Evaluation Metrics			
	DSC	MHD (mm)	PVKD (%)	
	Mean±SD	Mean±SD	Mean±SD	*P*-value
**Our method**	0.95_±0.01_	3.9_±0.76_	9.5_±2.2_	——
VLS [35]	0.90_±1.8_	5_±0.85_	10.8_±4.7_	<0.0001
RFC [42]	0.84_±1.43_	10.4_±3.4_	29.5_±2.1_	<0.0001
CV [31]	0.71_±11_	76_±12_	46_±13_	<0.0001

**Fig 9:**
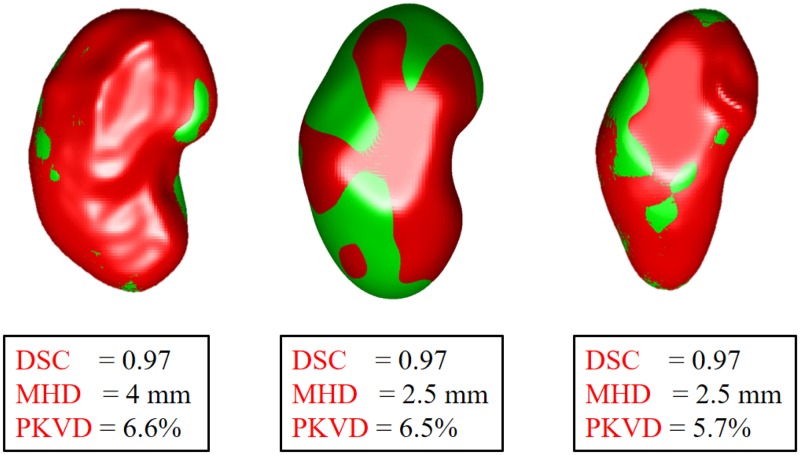
Our 3D segmentation (red) with respect to the expert’s manual ground truth (green) for three subjects with the associated accuracy scores.

**Fig 10:**
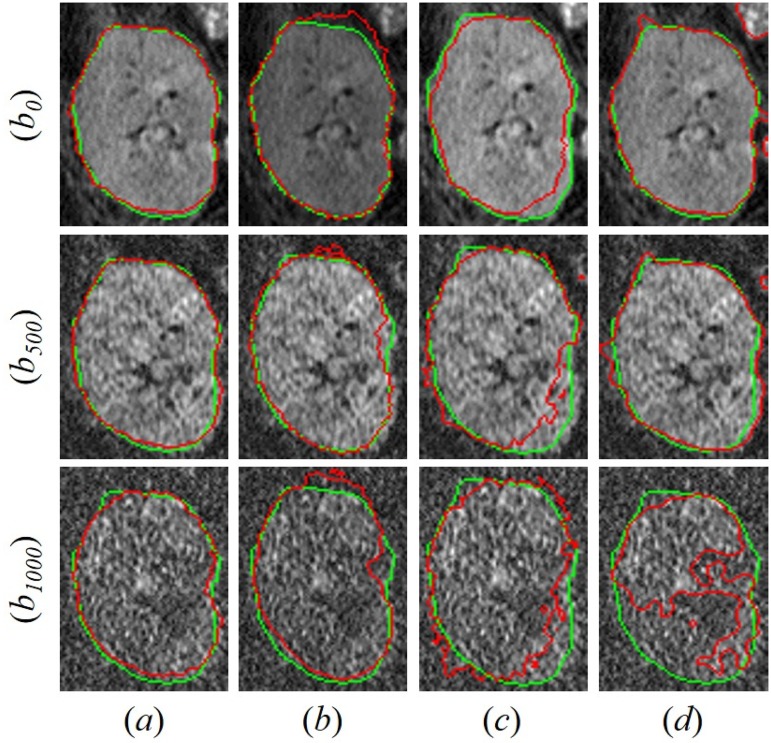
Comparative coronal cross-sectional segmentation results for the proposed approach (a), the vector level sets [35] (b), the segmentation using the Random Forest classifier [42] (c), and the traditional CV [31] level set (d) for one subjects at *b*_0_ (first row) and higher *b*_*i*_ values (*b*_500_ (second raw) and *b*_1000_ (third raw)). The model segmentation is shown in red with respect to the manual ground truth (green) from an expert.

Fig 9 shows 3D segmentation results for three different subjects with their associated evaluation metrics. In particular, the developed segmentation technique proved its ability to precisely segment the kidney, especially at higher *b*_*i*_ values. Shown in Fig 10, favorable comparative results between the proposed segmentation approach and the previously mentioned state-of-the-art segmentation methods for one subjects at *b*_0_ and higher *b*_*i*_ values (*b*_500_ and *b*_1000_), which also confirm the high accuracy and robustness of the proposed segmentation method to low contrast and noisy images.

### Performance of the proposed segmentation technique

After testing the proposed segmentation approach on the available DW-MRI datasets (a total of 64 subjects) in a leave-one-subject-out scenario, our system showed a notable better segmentation performance than the aforementioned well-known segmentation methods confirmed by the higher DSC of 0.95_±0.01_, lower MHD (mm) of 3.9_±0.76_ and lower PKVD (%) of 9.5_±2.2_.

Currently, the entire processing pipeline is implemented in MATLAB using a Dell Precision WorkStation T7500 (4 Intel Xeon W5590 CPUs (2 Cores/CPU) @ 3.33 GHz with 48 GB RAM and 4 TB RAID hard drive). For an input DW-MRI dataset of size 256 × 256 × 50 voxels of each *b*-value scan, the whole segmentation pipeline takes around 1.83 min. For processing all *b*-value scans and obtaining the final segmentation, the entire processing takes about 20.13 min on average.

### Clinical value of the contributions

Preliminary segmentation results outlined in this paper have shown that the proposed segmentation approach can segment kidneys from surrounding tissues with high reliability and accuracy. Taking into consideration that kidney segmentation is the first step in developing a fully automated CAD system for the early detection of acute renal transplant rejection, the more accurate the segmentation is, the more accurate estimating discriminatory features from the segmented kidneys will be. This will in turn be suitable for clinical applications to differentiate between non-rejection and acute rejection renal transplant.

Moreover, the proposed fully automated segmentation technique provides fast segmentation per *b*-value scan (in approximately 1.83 min with this MATLAB version) including all the pre-processing steps and the non-rigid registration, which is compatible with clinical routine. These abilities of the proposed segmentation technique encourages us to start developing a complete CAD system that will help clinicians to initiate timely interventions with appropriate treatments, which in turn can improve the delivery of healthcare in the USA and worldwide as a new, automated, fast, and relatively inexpensive diagnostic tool for early detection of acute renal transplant rejection.

## Conclusions and future work

In summary, this paper presented a segmentation technique is very promising to provide the guidance for the level-set geometric deformable model to accurately segment kidneys from the other abdomen structures and surrounding tissues using (3D + b-value) DW-MRI data. Our segmentation has been notably enhanced in terms of robustness to noise and low contrast, especially at higher *b*-values, and accuracy after adding the higher-order MGRF model combined with the adaptive shape model and the first-order visual appearance model. This can be explained in part by abilities of the higher-order MGRF model to capture more intricate inhomogeneities of grey levels between different structures, (e.g. cortex and medulla.) Moreover, the segmentation results hold promise to be incorporated in a complete CAD system for the early detection of acute renal transplant rejection, thus; the appropriate physiological parameters will be extracted from the segmented kidneys (e.g., apparent diffusion coefficient (ADC)) to be used as our future discriminatory features.

In the future, we plan to test the proposed segmentation technique on more datasets collected from different locations with different scanning parameters to prove the reliability of the presented technique in dealing with diverse datasets. In addition, we are currently working to improve and optimize the efficiency of the segmentation algorithm to reduce the total running time as recommended by our medical collaborator, by converting our codes to C++ or using the GPU. It is worth mentioning that we plan to investigate the effect of adding a 3D B-spline non-rigid registration step –after segmenting the kidneys– on handling the possible potential motion and image distortion between the individual *b*-values caused by eddy current and magnetic susceptibility changes, which might lead to a more accurate estimation of the ADCs to be used as our future discriminatory features between the acute rejection and non-rejection renal transplants. Furthermore, the presented segmentation technique can be extended to be applied on segmenting different soft tissues (e.g., prostate, liver, and spleen).

## References

[1] Center for Disease Control and Prevention, et al National Chronic Kidney Disease Fact Sheet, 2017 Atlanta, GA: US Department of Health and Human Services, 2017.

[2] Hollis E, Shehata M, Khalifa F, El-Ghar MA, El-Diasty T, El-Baz A. Towards non-invasive diagnostic techniques for early detection of acute renal transplant rejection: A review. The Egyptian Journal of Radiology and Nuclear Medicine. 2017;48(1):257–269. 10.1016/j.ejrnm.2016.11.005

[3] Abou-El-Ghar M, El-Diasty T, El-Assmy A, Refaie H, Refaie A, Ghoneim M. Role of diffusion-weighted MRI in diagnosis of acute renal allograft dysfunction: A prospective preliminary study. The British Journal of Radiology. 2014;85(1014):e206–e211. 10.1259/bjr/53260155 PMC3474109 22215880

[4] Liu G, Han F, Xiao W, Wang Q, Xu Y, Chen J. Detection of renal allograft rejection using blood oxygen level-dependent and diffusion weighted magnetic resonance imaging: A retrospective study. BMC Nephrology. 2014;15(1):158 10.1186/1471-2369-15-158 25270976 PMC4192395

[5] Khalifa F, Beache GM, El-Ghar MA, El-Diasty T, Gimel’farb G, Kong M, et al Dynamic Contrast-Enhanced MRI-Based Early Detection of Acute Renal Transplant Rejection. IEEE Transaction on Medical Imaging. 2013;32(10):1910–1927. 10.1109/TMI.2013.2269139 23797240

[6] Park SY, Kim CK, Park BK, Kim SJ, Lee S, Huh W. Assessment of early renal allograft dysfunction with blood oxygenation level-dependent MRI and diffusion-weighted imaging. European Journal of Radiology. 2014;83(12):2114–2121. 10.1016/j.ejrad.2014.09.017 25452096

[7] Wypych-Klunder K, Adamowicz A, Lemanowicz A, Szczȩsny W, Włodarczyk Z, Serafin Z. Diffusion-weighted MR imaging of transplanted kidneys: Preliminary report. Polish Journal of Radiology. 2014;79:94–98. 10.12659/PJR.890502 24826200 PMC4018246

[8] Mackelaite L, Ouseph R, El-Baz A, Gaweda A. Cortical CT Perfusion of the Live Donor Kidneys as a Predictor of Post Transplant Graft Function. In: American Journal of Transplantation. vol. 12; 2012 p. 329–329.

[9] Steiger P, Barbieri S, Kruse A, Ith M, Thoeny HC. Selection for biopsy of kidney transplant patients by diffusion-weighted MRI. European Radiology. 2017;27(10):4336–4344. 10.1007/s00330-017-4814-z 28374076

[10] Hueper K, Khalifa AA, Bräsen JH, Chieu V, Dai V, Gutberlet M, et al Diffusion-Weighted imaging and diffusion tensor imaging detect delayed graft function and correlate with allograft fibrosis in patients early after kidney transplantation. Journal of Magnetic Resonance Imaging. 2016;44(1):112–121. 10.1002/jmri.25158 26778459

[11] Giele E, De Priester J, Blom J, Den Boer J, Van Engelshoven J, Hasman A, et al Movement correction of the kidney in dynamic MRI scans using FFT phase difference movement detection. Journal of Magnetic Resonance Imaging. 2001;14(6):741–749. 10.1002/jmri.10020 11747031

[12] Sun Y. Registration and segmentation in perfusion MRI: Kidneys and hearts. PhD dissertation, Carnegie Mellon University: Pittsburg; 2004.

[13] Mavromatis S, Boi JM, Sequeira J. Medical image segmentation using texture directional features. In: Engineering in Medicine and Biology Society, 2001. vol. 3. IEEE; 2001. p. 2673–2676.

[14] de Priester JA, Kessels AG, Giele EL, den Boer JA, Christiaans MH, Hasman A, et al MR renography by semiautomated image analysis: performance in renal transplant recipients. Journal of Magnetic Resonance Imaging. 2001;14(2):134–140. 10.1002/jmri.1163 11477671

[15] Giele ELW. Computer methods for semi-automatic MR renogram determination. Research thesis, Technische Universiteit Eindhoven; 2002.

[16] Koh H, Shen W, Shuter B, Kassim AA. Segmentation of kidney cortex in MRI studies using a constrained morphological 3D H-maxima transform. In: IEEE International Conference on Control, Automation, Robotics and Vision. ICARCV’06. IEEE; 2006. p. 1–5.

[17] Pohle R, Toennies KD. A new approach for model-based adaptive region growing in medical image analysis. In: International Conference on Computer Analysis of Images and Patterns; 2001. p. 238–246.

[18] Boykov Y, Funka-Lea G. Graph cuts and efficient ND image segmentation. International journal of computer vision. 2006;70(2):109–131. 10.1007/s11263-006-7934-5

[19] Rusinek H, Boykov Y, Kaur M, Wong S, Bokacheva L, Sajous JB, et al Performance of an automated segmentation algorithm for 3D MR renography. Magnetic Resonance in Medicine. 2007;57(6):1159–1167. 10.1002/mrm.21240 17534915

[20] Ali A, Farag A, El-Baz A. Graph cuts framework for kidney segmentation with prior shape constraints. Medical Image Computing and Computer-Assisted Intervention—MICCAI 2007. 2007; p. 384–392. 10.1007/978-3-540-75757-3_47 18051082

[21] Chevaillier B, Mandry D, Collette JL, Claudon M, Galloy MA, Pietquin O. Functional segmentation of renal DCE-MRI sequences using vector quantization algorithms. Neural processing letters. 2011;34(1):71–85. 10.1007/s11063-011-9184-y

[22] Freiman M, Kronman A, Esses SJ, Joskowicz L, Sosna J. Non-parametric iterative model constraint graph min-cut for automatic kidney segmentation. In: International Conference on Medical Image Computing and Computer-Assisted Intervention; vol. 6363; 2010. p. 73–80. 10.1007/978-3-642-15711-0_10 20879385

[23] Li S, Zöllner FG, Merrem AD, Peng Y, Roervik J, Lundervold A, et al Wavelet-based segmentation of renal compartments in DCE-MRI of human kidney: Initial results in patients and healthy volunteers. Computerized Medical Imaging and Graphics. 2012;36(2):108–118. 10.1016/j.compmedimag.2011.06.005 21704499

[24] Yang X, Ghafourian P, Sharma P, Salman K, Martin D, Fei B. Nonrigid registration and classification of the kidneys in 3D dynamic contrast enhanced (DCE) MR images. In: Proceedings of SPIE. vol. 8314; 2012. p. 83140B. 10.1117/12.912190 PMC3314431 22468206

[25] Leventon ME, Grimson WEL, Faugeras O. Statistical shape influence in geodesic active contours. In: Proceedings of IEEE Conference on Computer Vision and Pattern Recognition. vol. 1; 2000. p. 316–323.

[26] Wang X, He L, Wee W. Deformable contour method: a constrained optimization approach. International Journal of Computer Vision. 2004;59(1):87–108. 10.1023/B:VISI.0000020672.14006.ad

[27] Tsagaan B, Shimizu A, Kobatake H, Miyakawa K, Hanzawa Y. Segmentation of kidney by using a deformable model. In: IEEE International Conference on Image Processing. vol. 3; 2001. p. 1059–1062.

[28] Sun Y, Moura JM, Ho C. Subpixel registration in renal perfusion MR image sequence. In: IEEE International Symposium on Biomedical Imaging: Nano to Macro. IEEE; 2004. p. 700–703.

[29] Song T, Lee VS, Rusinek H, Kaur M, Laine AF. Automatic 4-D registration in dynamic MR renography. In: IEEE International Conference of the Engineering in Medicine and Biology Society (EMBS’06). IEEE; 2006. p. 3067–3070. 10.1109/IEMBS.2005.1617122 17282891

[30] Sun Y, Jolly MP, Moura J. Integrated registration of dynamic renal perfusion MR images. In: IEEE International Conference on Image Processing. ICIP’04. vol. 3; 2004. p. 1923–1926.

[31] Chan TF, Vese L. Active contours without edges. IEEE Transactions on Image Processing. 2001;10(2):266–277. 10.1109/83.902291 18249617

[32] Kim S. A hybrid level set approach for efficient and reliable image segmentation. In: IEEE International Symposium on Signal Processing and Information Technology; 2005. p. 743–748.

[33] Lie J, Lysaker M, Tai XC. A binary level set model and some applications to Mumford-Shah image segmentation. IEEE Transactions on Image Processing. 2006;15(5):1171–1181. 10.1109/TIP.2005.863956 16671298

[34] Yan P, Kassim AA, Shen W, Shah M. Modeling interaction for segmentation of neighboring structures. IEEE Transactions on Information Technology in Biomedicine. 2009;13(2):252–262. 10.1109/TITB.2008.2010492 19171526

[35] El Munim HEA, Farag AA. Curve/surface representation and evolution using vector level sets with application to the shape-based segmentation problem. IEEE Transactions on Pattern Analysis and Machine Intelligence. 2007;29(6):945–958. 10.1109/TPAMI.2007.1100 17431295

[36] Abdelmunim H, Farag AA, Miller W, AboelGhar M. A kidney segmentation approach from DCE-MRI using level sets. In: IEEE Computer Society Conference on Computer Vision and Pattern Recognition Workshops, CVPRW’08; 2008. p. 1–6.

[37] Spiegel M, Hahn DA, Daum V, Wasza J, Hornegger J. Segmentation of kidneys using a new active shape model generation technique based on non-rigid image registration. Computerized Medical Imaging and Graphics. 2009;33(1):29–39. 10.1016/j.compmedimag.2008.10.002 19046849

[38] Yuksel SE, El-Baz A, Farag AA, El-Ghar M, Eldiasty T, Ghoneim MA. A kidney segmentation framework for dynamic contrast enhanced magnetic resonance imaging. Journal of Vibration and Control. 2007;13(9-10):1505–1516. 10.1177/1077546307077417

[39] El-Baz A, Elnakib A, Khalifa F, El-Ghar MA, McClure P, Soliman A, et al Precise segmentation of 3-D magnetic resonance angiography. IEEE Transactions on Biomedical Engineering. 2012;59(7):2019–2029. 10.1109/TBME.2012.2196434 22547453

[40] Campadelli P, Casiraghi E, Pratissoli S. A segmentation framework for abdominal organs from CT scans. Artificial Intelligence in Medicine. 2010;50(1):3–11. 10.1016/j.artmed.2010.04.010 20542673

[41] Gloger O, Tonnies KD, Liebscher V, Kugelmann B, Laqua R, Volzke H. Prior shape level set segmentation on multistep generated probability maps of MR datasets for fully automatic kidney parenchyma volumetry. IEEE Transactions on Medical Imaging. 2012;31(2):312–325. 10.1109/TMI.2011.2168609 21937343

[42] Cuingnet R, Prevost R, Lesage D, Cohen LD, Mory B, Ardon R. Automatic detection and segmentation of kidneys in 3D CT images using random forests. In: International Conference on Medical Image Computing and Computer-Assisted Intervention; vol. 7512; 2012. p. 66–74. 10.1007/978-3-642-33454-2_9 23286115

[43] Tustison NJ, Avants BB, Cook PA, Zheng Y, Egan A, Yushkevich PA, et al N4ITK: Improved N3 bias correction. IEEE Transactions on Medical Imaging. 2010;29(6):1310–1320. 10.1109/TMI.2010.2046908 20378467 PMC3071855

[44] Rudra A, Chowdhury A, Elnakib A, Khalifa F, Soliman A, Beache GM, et al Kidney segmentation using graph cuts and pixel connectivity. Pattern Recognition Letters. 2013;34(13):1470–1475. 10.1016/j.patrec.2013.05.013

[45] Jensen HG, Lauze F, Nielsen M, Darkner S. Locally Orderless Registration for Diffusion Weighted Images. In: Proc. International Conference on Medical Image Computing and Computer-Assisted Intervention, (MICCAI’15) (Lecture Notes in Computer Science); vol. 9350; 2015. p. 305–312.

[46] Yap PT, Zhang Y, Shen D. Brain Tissue Segmentation Based on Diffusion MRI Using l0 Sparse-Group Representation Classification. In: Medical Image Computing and Computer-Assisted Intervention, (MICCAI’15); vol. 9351; 2015 p. 132–139. PMC6054460 30035276

[47] Balafar MA, Ramli AR, Saripan MI, Mashohor S. Review of brain MRI image segmentation methods. Artificial Intelligence Review. 2010;33(3):261–274. 10.1007/s10462-010-9155-0

[48] Liu X, Langer D, Haider M, Van der Kwast T, Evans A, Wernick M, et al. Unsupervised segmentation of the prostate using MR images based on level set with a shape prior. In: Proc. Annual International Conference of the IEEE, Engineering in Medicine and Biology Society, (EMBC’09); 2009. p. 3613–16. 10.1109/IEMBS.2009.5333519 19964082

[49] Osher S, Fedkiw R. Level Set Methods and Dynamic Implicit Surfaces. New York, USA: Springer Verlag; 2006.

[50] Farag A, El-Baz A, Gimelfarb G. Precise segmentation of multi-modal images. IEEE Transactions on Image Processing. 2006;15(4):952–968. 10.1109/TIP.2005.863949 16579381

[51] Glocker B, Komodakis N, Paragios N, Navab N. Non-rigid registration using discrete MRFs: Application to thoracic CT images. In: Proc. MICCAI Workshop on Evaluation of Methods for Pulmonary Image Registration, (MICCAI’10); 2010. p. 147–154.

[52] Dice LR. Measures of the amount of ecologic association between species. Ecology. 1945;26(3):297–302. 10.2307/1932409

[53] Gerig G, Jomier M, Chakos M. Valmet: A new validation tool for assessing and improving 3D object segmentation. In: Proc. International Conference on Medical Image Computing and Computer Assisted Intervention, (MICCAI’01); vol. 2208; 2001. p. 516–523.

[54] Shehata M, Khalifa F, Soliman A, Alrefai R, El-Ghar MA, Dwyer AC, et al. A novel framework for automatic segmentation of kidney from DW-MRI. In: Proc. IEEE 12th International Symposium on Biomedical Imaging, (ISBI’15); 2015. p. 951–954.

[55] Shehata M, Khalifa F, Soliman A, Alrefai R, El-Ghar MA, Dwyer AC, et al. A level set-based framework for 3D kidney segmentation from diffusion MR images. In: Proc. IEEE 22nd International Conference on Image Processing, (ICIP’15); 2015. p. 4441–4445.

